# Heterogeneous Structure, Mechanisms of Counterion Exchange, and the Spacer Salt Effect in Complex Molten Salt Mixtures Including LaCl_3_

**DOI:** 10.1021/acs.jpcb.4c01429

**Published:** 2024-04-16

**Authors:** Matthew S. Emerson, Alexander S. Ivanov, Leighanne C. Gallington, Dmitry S. Maltsev, Phillip Halstenberg, Sheng Dai, Santanu Roy, Vyacheslav S. Bryantsev, Claudio J. Margulis

**Affiliations:** † Department of Chemistry, The University of Iowa, Iowa City, Iowa 52242, United States; ‡ Chemical Sciences Division, Oak Ridge National Laboratory, Oak Ridge, Tennessee 37831, United States; § X-ray Science Division, Argonne National Laboratory, Argonne, Illinois 60439, United States; ∥ Department of Chemistry, University of Tennessee, Knoxville, Tennessee 37996, United States

## Abstract

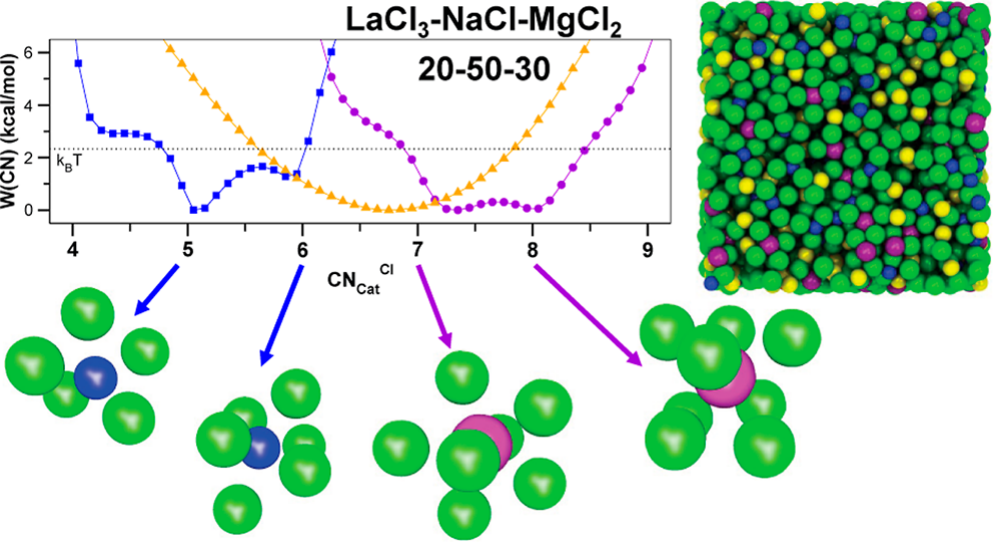

Complex molten chloride salt mixtures of uranium, magnesium, and sodium are top candidates for promising nuclear energy technologies to produce electricity based on molten salt reactors. From a local structural perspective, LaCl_3_ is similar to UCl_3_ and hence a good proxy to study these complex salt mixtures. As fission products, lanthanide salts and their mixtures are also very important in their own right. This article describes from an experimental and theory perspective how very different the structural roles of MgCl_2_ and NaCl are in mixtures with LaCl_3_. We find that, whereas MgCl_2_ becomes an integral part of multivalent ionic networks, NaCl separates them. In a recent article (*J. Am. Chem. Soc.***2022**, *144*, 21751–21762) we have called the disruptive behavior of NaCl “the spacer salt effect”. Because of the heterogeneous nature of these salt mixtures, there are multiple structural motifs in the melt, each with its particular free energetics. Our work identifies and quantifies these; it also elucidates the mechanisms through which Cl^–^ ions exchange between Mg^2+^-rich and La^3+^-rich environments.

## 1 Introduction

Molten salt mixtures of the actinides or lanthanides are becoming quite useful for industry and in the energy sector. For example, UCl_3_–NaCl–MgCl_2_ and PuCl_3_–NaCl–MgCl_2_ are promising molten salt fast reactor fuel candidates,^1,2^ LaCl_3_–KCl–MgCl_2_ mixtures are used to synthesize various LaMg alloys^3^ used as tunable mirrors,^4^ and various Cl^–^-based mixture melts have been studied in the context of corrosion.^5−9^ It is also important to have fast database access to thermodynamic properties of complex binary and ternary salt combinations.^10−15^ There is a direct link between structural and thermodynamic studies such as ours and the quasi-thermodynamic models that are at the core of such databases since free-energetic properties in those are modeled based on the coordination number of the ions.^16−18^ Of course, in reality it was found^19−27^ that, at the temperatures at which salts are in the molten state, cations have an ensemble of interconverting coordination structures that are temperature dependent. For multivalent cations in combination with chloride, a significantly polarizable anion, there are further complexities. One would intuitively think that, due to Coulombic repulsion, multipositive ions would want to stay apart, each forming its own distinct “complex” unit with counterions. Yet, this could not be further from what actually occurs; multivalent cations tend to be closer than expected and form networks bridged by the highly polarizable chloride counterions. Whereas this is true in general,^19,22,28−37^ the shape of these networks and the coordination number distribution very much depend on the actual identity of the cations.

A fascinating phenomenon we discussed in a recent article^22^ is the disruptive effect of low valency cations such as Na^+^ on multivalent salt structure. It is not a stretch of the imagination to think of NaCl the way one thinks of the apolar domains in room-temperature ionic liquids (ILs).^38−52^ In the case of ILs one commonly finds strands of positive–negative charge alternation “spaced” by apolar domains. The smoking gun in such systems is the appearance of a scattering prepeak or first sharp diffraction peak linked with the spacing between charge strands when these are separated by an apolar domain.^39,40,44,53^ NaCl does just this when mixed with LaCl_3_;^22^ it partitions the La^3+^ networks, thereby causing the appearance of a new low-q scattering peak that is absent in both neat LaCl_3_ and NaCl. We call this the spacer salt effect. An important question from the point of view of the formation of networks and the coordination structure of La^3+^ is whether other cations such as Mg^2+^ act similarly to Na^+^. Is the small and hard Mg^2+^ ion also a disrupting spacer when combined with LaCl_3_? And, what is the structure of complex ternary cationic mixtures? Zhao et al. studied the structure of molten NaCl–KCl–MgCl_2_–LaCl_3_ mixtures using machine learning molecular dynamics (MD) and found that what they referred to as “La^3+^ clusters” were disrupted with increasing MgCl_2_ concentration.^54^ Whereas this is likely, we make the case that MgCl_2_ is very different from NaCl and not a spacer salt.

## 2 Results and Discussion

**Figure 1:**
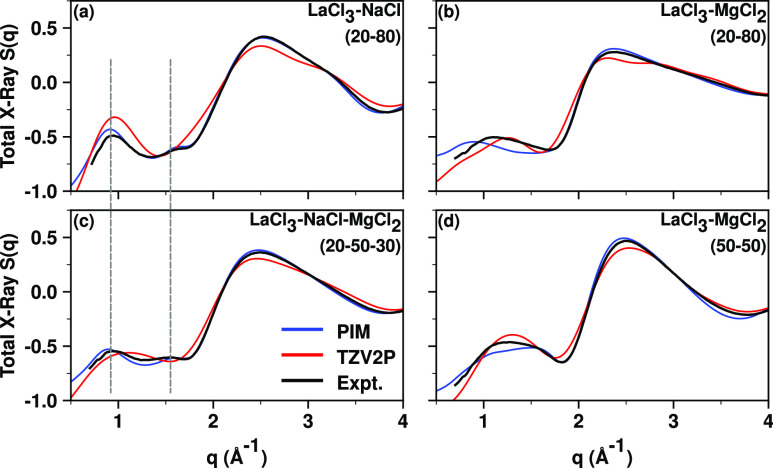
Comparison of structure function S(q) at 900 °C for LaCl_3_–MgCl_2_, LaCl_3_–NaCl, and LaCl_3_–NaCl–MgCl_2_ mixtures with mol % denoted in figure titles. Notice how the experimental S(q) has two peaks below 2 Å^–1^ when NaCl is present in the melt; these are highlighted with dashed vertical lines to guide the eye in subfigures (a) and (c). S(q) is plotted up to 4 Å^–1^ to highlight important interionic features; Figure S1 shows S(q) in its full range. PIM and experimental S(q) for subfigure (a) use the same data as that in ref (22).

We start by highlighting the obvious qualitative difference between experimental X-ray structure functions, S(q), for LaCl_3_ melts containing a significant fraction of NaCl—a spacer salt—in contrast to those in which the spacer salt is absent. High-energy X-ray scattering measurements at 900 °C were performed at beamline 11-ID-B of the Advanced Photon Source (APS) from which S(q) functions, shown in their full q-range in Figure S1, were obtained following protocols described in section S.1. The experimental S(q) for LaCl_3_–NaCl (20–80 mol %) and LaCl_3_–NaCl–MgCl_2_ (20–50–30 mol %), black line in Figure 1a,c, shows two distinct peaks in the regime below 2 Å^–1^. The peak close to 1.5 Å^–1^ is what we have called in multiple prior publications a charge alternation peak^22−24,36−42,55−57^ and will be discussed in a subsequent section. Instead, the feature below ∼1.1 Å^–1^ is a prepeak that is absent for neat LaCl_3_ and NaCl; the prepeak only appears when these are mixed.^22^ The two peaks are highlighted in Figure 1a,c with dashed vertical lines to guide the eye. Notice that, when NaCl is absent such as in the 50%–50% and 20%–80% mixtures of LaCl_3_ with MgCl_2_ in Figure 1b,d, only a broad feature is observed in the same q-range below 2 Å^–1^.

We have simulated multiple salt mixture melts using both the polarizable ion model (PIM) and *ab initio* molecular dynamics (AIMD). Technical details for all our theory and computational protocols are given in section S.1. Whereas both AIMD and the PIM do quite well across the board when compared with the experimental scattering results measured at the APS and reported in Figure S1, it is clear that, either due to finite size effects (AIMD simulations include many fewer ions) or deficiencies at the DFT level of theory for both shorter- and longer-range interactions, the PIM does better overall; furthermore, the PIM model for LaCl_3_ was originally fit to better reproduce structure.^31,32,58^ Based on this, analysis in the main text will be based on the PIM and, when appropriate, AIMD results will be provided in the Supporting Information for comparison. What is important is that the PIM captures the two peaks below 2 Å^–1^ for mixtures including NaCl but AIMD does not always do so, as can be gleaned from Figure 1a,c. None of the simulation techniques provide a perfect experimental match in the low-q regime for systems containing only LaCl_3_ and MgCl_2_.

**Figure 2:**
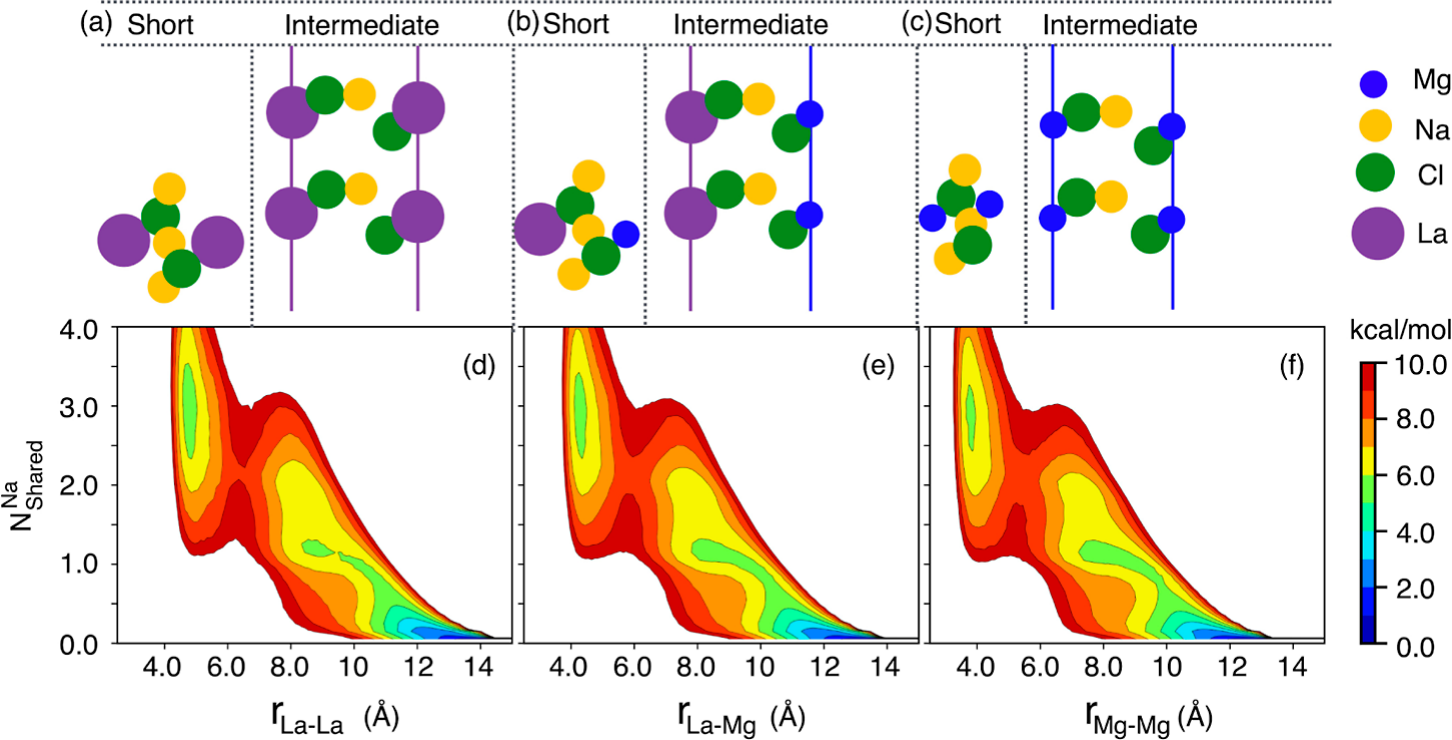
For the LaCl_3_–NaCl–MgCl_2_ (20–50–30 mol %) ternary mixture, (a) through (c), schemes depicting short- and intermediate-range structural motifs associated with the free energies as a function of La–La, La–Mg, and Mg–Mg separation distances and the number of shared Na^+^ ions between them plotted in subfigures (d) through (f). Results presented here are for the PIM; corresponding results from AIMD are shown in Figure S2.

From a practical perspective and before we dive deeper into our analysis, we want to explain in real space the role played by the spacer salt when compared to MgCl_2_  which is not a spacer salt. We start to understand the intricate structure of a ternary mixture such as LaCl_3_–NaCl–MgCl_2_ (20–50–30 mol %), when looking at free energy landscapes and accompanying ionic schemes depicted in Figure 2. We will see from the analysis presented below that multivalent cations form chloride-decorated aggregates or networks. We can intuitively think of these aggregates as having −La–La–, −La–Mg–, and −Mg–Mg– minimal subunits. The aggregates have multiple such subunits and branches that lead to what are commonly described as polyhedra. If there is a low valency salt in the mixture such as NaCl, this salt acts as a spacer that separates the aggregates. Figure 2 shows how Na^+^ ions structurally correlate and free energetically interact with La^3+^ and Mg^2+^ (Figure S2 shows similar data from AIMD). As a function of the separation distance between the multivalent cations there are different minima characterized by the number of Na^+^ ions spacing them or shared by them—about 3 Na^+^ for the first minimum, between 1 and 1.5 for the second, and none when the multivalent cations are too far apart to share Na^+^ ions. Pay particular attention to the second minimum at “intermediate distance” for which between 1 and 1.5 Na^+^ ions are shared by multivalent cation pairs at a separation of about 8 Å. The set of structural motifs labeled “intermediate” in the top portion of Figure 2 corresponding to the second free energy minimum are the ones associated with “intermediate range order” and the scattering prepeak in Figure 1c. Similar structural patterns corresponding to intermediate range order exist for the binary mixture in Figure 1a, as can be gleaned from Figure 6 in ref (22). In the schemes atop Figure 2, the charge network is pictorially represented with solid vertical purple or blue lines to guide the eye. This is not meant to imply that a network only has one type of multivalent cation or that there is no branching; instead all three −La–La–, −La–Mg–, and −Mg–Mg– patterns are present. Apparent from the almost identical nature of the 2D free energy surfaces in Figure 2d–f is that the structural correlations between Na^+^ ions with Mg^2+^ or La^3+^ ions are very similar.

### 2.1 Both the Prepeak and the Charge Alternation Peak are Due to Alternation

**Figure 3:**
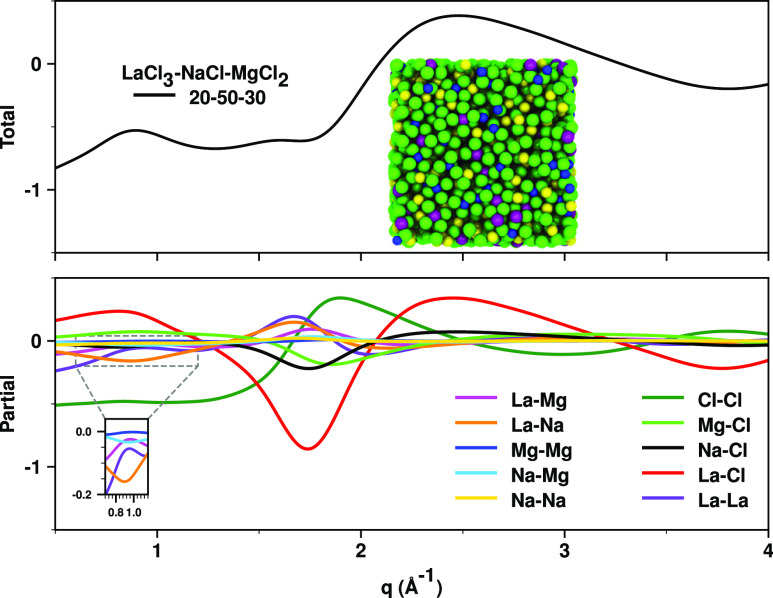
Upper panel: Total X-ray S(q) for the LaCl_3_–NaCl–MgCl_2_ (20–50–30 mol %) mixture melt and a snapshot of the PIM simulation box with ions color-coded as in Figure 2. Bottom panel: The partial subcomponents of S(q); an inset shows the behavior of selected subcomponents in the prepeak region associated with the “spacer salt effect”. Both in the prepeak region (at or below ∼1.1 Å^–1^) and in the charge alternation region (∼1.5–2 Å^–1^) there are patterns of peaks and antipeaks that define distinct types of alternations. The prepeak is associated with free energy minima and accompanying ionic schemes defined as “Intermediate” in Figure 2, whereas the charge alternation features are associated with the prominent minima at shorter range in Figure 4.

We remind the readers that peaks and antipeaks at similar q-values in the partial subcomponents of S(q) are the hallmark of liquid phase structural alternation^22−24,36−46,56,59^ and ask them to keep their focus on the prepeak region below 1.1 Å^–1^ in Figure 1a,c. The partial subcomponents of S(q) are shown in the bottom panel of Figure 3 with an inset highlighting the behavior of selected subcomponents in the prepeak region. Notice from the inset that La–Na correlations as well as Na–Mg correlations are antipeaks (point down), whereas La–La, La–Mg, and Mg–Mg correlations are peaks. The peaks imply enhanced probability of multivalent cations at a distance associated with this q-value; this distance is what we referred to as “intermediate range” in Figure 2. The antipeaks at the same q-value imply a depletion of probability of finding Na^+^ at this same distance from the multivalent cations; in other words, this is where we do not find Na^+^ ions. The enhancements of probability at ∼0.7–1.1 Å^–1^ in Figure 3 occur because of the second free energy minimum between the multivalent cations at intermediate range around 8 Å in subpanels of Figure 2. The depletion of probability for finding Na^+^ at this distance from a multivalent cation is because, as a component of the spacer salt, it is often found at about half this distance. In other words, *there is alternation between the multivalent cations and the spacer salt*.

Now that we understand that the intermediate range behavior in Figure 2 and the peaks and antipeaks associated with the prepeak in Figure 3 are all manifestations of the same spacer salt effect, we move on to explain charge alternation associated with the peaks and antipeaks in the region between ∼1.5–2 Å^–1^; we also briefly mention adjacency correlations.^22−24,36,37,39,56^ The dominant contributions to the overall S(q) in the top panel of Figure 3 beyond 2 Å^–1^ are from correlations between ions that are adjacent, are at significant concentration, and have sufficient X-ray scattering power. These include the tail on the high-q side of the Cl–Cl peak at about 2 Å^–1^ and, most importantly, the La–Cl peak at higher q-values in the bottom panel of Figure 3. A thorough explanation of these simple adjacency correlations has been provided in our recent paper,^22^ and we write no more about them here.

From the bottom panel in Figure 3, in the region between 1.5 and 2 Å^–1^ we see a busy pattern of peaks and antipeaks. Notice for example that La–Mg, Mg–Mg, La–La, La–Na, and Cl–Cl contributions all show as peaks and that these are all from same-charge-sign ions (i.e., no +/– contributions). Instead, the La–Cl, Na–Cl, and Mg–Cl contributions to S(q) in the 1.5–2 Å^–1^ regime are all antipeaks. In other words, there should be a statistical depletion of cation–anion pairs at the expected distance between two cations. Why? Because the anions are in between the two cations due to charge alternation. Obviously, the distance between cations differs by the type of cation; this is why each cation–cation contribution in this region peaks at a different q-value.

**Figure 4:**
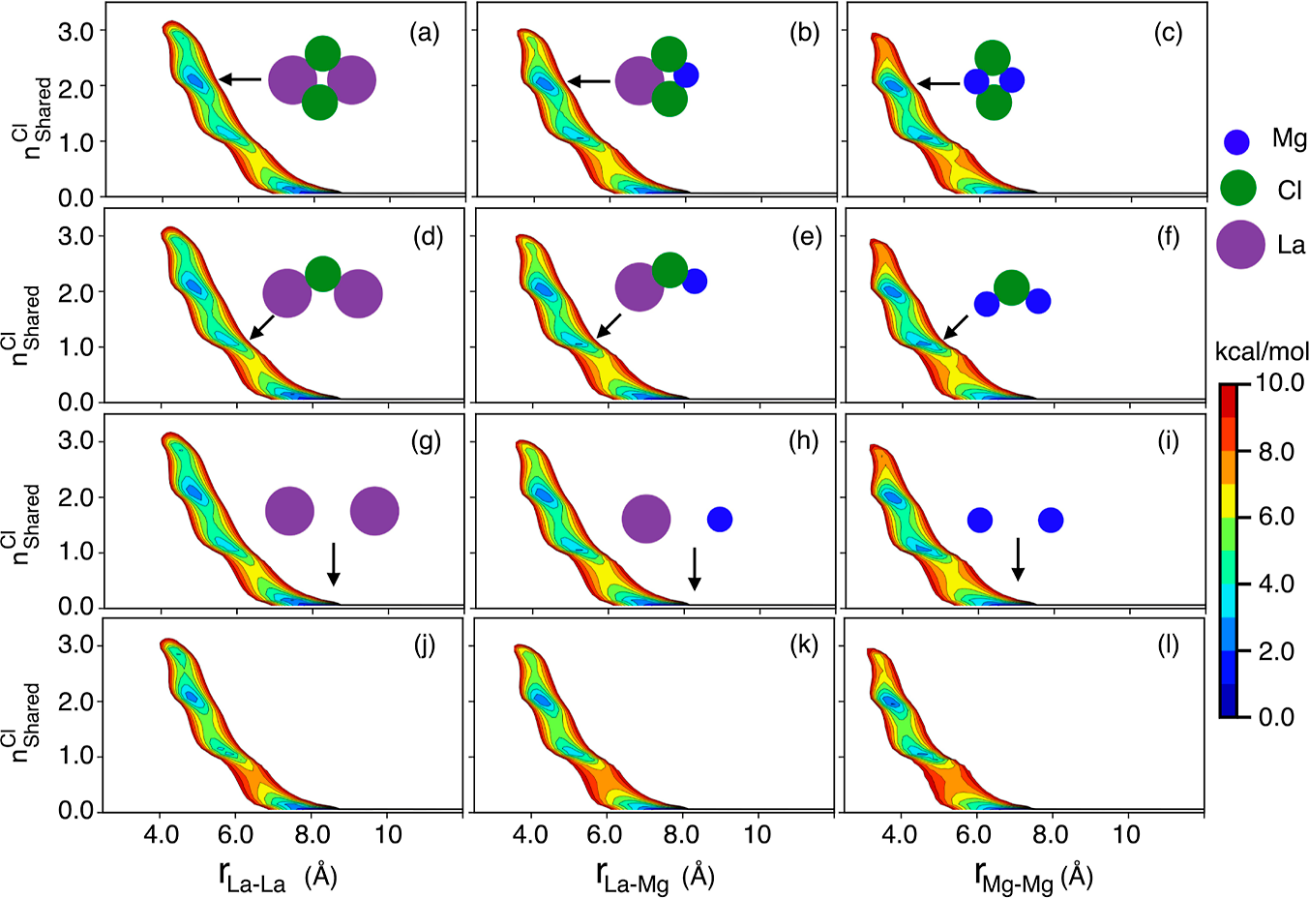
For 20 mol % (a–c), 30 mol % (d–f), and 50 mol % (g–i) LaCl_3_ in binary LaCl_3_–MgCl_2_ mixture melts and for 20 mol % (j–l) LaCl_3_ in the ternary LaCl_3_–NaCl–MgCl_2_ melt; 2D free energy surfaces as a function of the number of shared chlorides between multivalent cations and their corresponding distance. Results presented here are for the PIM; corresponding results from AIMD are shown in Figure S3.

How do these charge alternation ionic patterns look in real space? And what is the free energy associated with them? For a variety of systems including binary and ternary mixtures, Figure 4 shows the free energy as a function of the distance between multivalent cations and as a function of the number of shared anions. As an example, Figure 4j shows free energy minima between ∼4 and 5 Å for our ternary mixture associated with two La^3+^ ions sharing two or three Cl^–^ counterions. The exact location of these first minima, associated with the charge alternation peak in the partial subcomponents of S(q), depends on the size of the cations. Notice in general how when two La^3+^ ions are at close distance they can accommodate three Cl^–^ counterions; because Mg^2+^ is smaller and less polarizing, this arrangement is not favored between two Mg^2+^ ions.

### 2.2 From the Bottom Up, Coordination Environments and Ion Networks

So far, we have discussed ionic patterns that give rise to the features in the partial subcomponents of S(q). Yet, it is clear that such patterns are only the tip of the iceberg. In subsequent sections we discuss how complex salts of multivalent cations are made of intricate networks in which the cations have multiple possible coordination states. In fact, it will become apparent that the ensemble of coordination states for a given species is not independent of that of others.

#### 2.2.1 Larger Concentration of La^3+^ Shifts to Higher Values the Cl^–^ Coordination of All Cations

**Figure 5:**
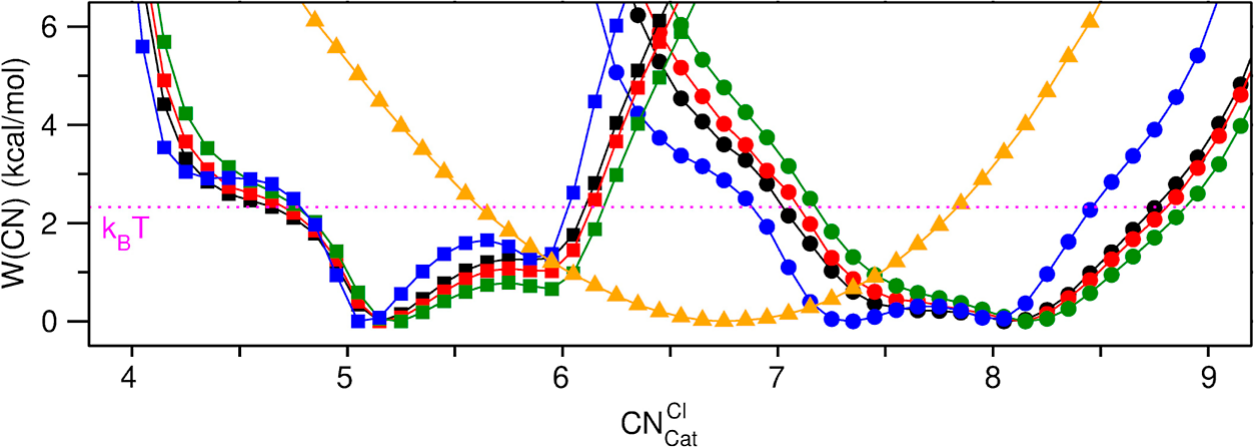
For all cations, free energy as a function of their Cl^–^ coordination number. These are depicted with circles for La^3+^, squares for Mg^2+^, and triangles for Na^+^. Different colors indicate different concentrations; specifically, in binary LaCl_3_–MgCl_2_ mixtures black is for 20 mol % LaCl_3_, red is for 30 mol % LaCl_3_, and green is for 50 mol % LaCl_3_. In the ternary mixture, which is 20 mol % in LaCl_3_, free energies for La^3+^ and Mg^2+^ are shown in blue and that for Na^+^ is shown in yellow. The thermal energy, which is high compared to several of the free energy minima (*k*_*B*_*T* = 2.33 kcal/mol), is indicated with a horizontal magenta line. Results presented here are for the PIM; corresponding plots from AIMD simulations can be found in Figure S4.

Figure 5 shows that, at a given temperature and melt composition, there are multiple minima in the free energy as a function of the chloride coordination number of the multivalent cations. Furthermore, the ensemble of coordination numbers for the multivalent cations is clearly concentration dependent. A different way of looking at this is that the coordination distribution of one species is coupled to that of other species. For example, the presence of La^3+^ cations increases the coordination number of the lower-valent Mg^2+^ cations in mixture melts. We attribute this phenomenon to the polarizing effect of La^3+^ on Cl^–^ allowing more of the anions to coordinate Mg^2+^. We found this already in a previous study^25^ on the effect of dilute Cr^3+^, a different trivalent cation, in a MgCl_2_–KCl melt. The reader is reminded that, for the most part, this is not the coordination of a “complex”, but instead, a single Cl^–^ coordinates with more than one cation.

In our mixture melts, the most likely coordination number of Mg^2+^ is approximately 5, but given the high thermal energy, states with  = 6 (more often) and  = 4 (less often) are also visited. The previously described effect of an increase in coordination of Mg^2+^ upon a larger concentration of La^3+^ can be seen from the fact that the likelihood of  = 6 increases and that of  = 4 decreases with an increase of La^3+^ concentration. The effect is the same for Na^+^; its chloride coordination number is close to 7 in the ternary 20:50:30 LaCl_3_:NaCl:MgCl_2_ mixture, while it is close to 6 in neat NaCl.^23^ Likewise, the coordination of K^+^ is around 6 in the neat KCl melt,^23^ but we found it to be around 9 in a CrCl_3_–MgCl_2_–KCl mixture.^25^

Interestingly, the effect extends also to the coordination of La^3+^, given that an increase in its concentration concomitantly results in an increase in its coordination number. As an example, in our binary LaCl_3_–MgCl_2_ mixtures at 50 mol % in each of the components, we find that, while making frequent almost barrierless transitions to 7 (quantified as 6.5 <  < 7.5), the most likely coordination for La^3+^ is 8 (defined as 7.5 <  < 8.5).  = 7 becomes more available at lower concentrations of LaCl_3_. The effect is not unique to La^3+^ as we have seen this also for other multivalent cations such as Mg^2+^.^37^ The result is also consistent with trends in the Cl^–^ coordination of La^3+^ in mixtures with NaCl.^22^ Notice that, in the ternary 20:50:30 LaCl_3_:NaCl:MgCl_2_ mixture,  = 7 and  = 8 are equally probable, which is again attributed to the lower mol % of LaCl_3_. Figure S4 shows coordination number results based on AIMD. Trends are similar to those observed for the PIM, but the actual coordination numbers for La^3+^ are shifted downward and those for Na^+^ are shifted upward. Because S(q) is better reproduced by the PIM (see Figure 1 and Figure S1), we believe that coordination ensembles derived from this technique are more accurate.

#### 2.2.2 Networks and Aggregates

In molten salts that include anion-polarizing multivalent cations such as Mg^2+^, Zn^2+^, and La^3+^, it is common to observe Cl^–^-decorated cationic aggregates and networks.^22,36,37,60,61^ These networks can have intricate topologies influenced by the concentration of companion salts that are network disrupting, such as the salts of monovalent cations.

**Figure 6:**
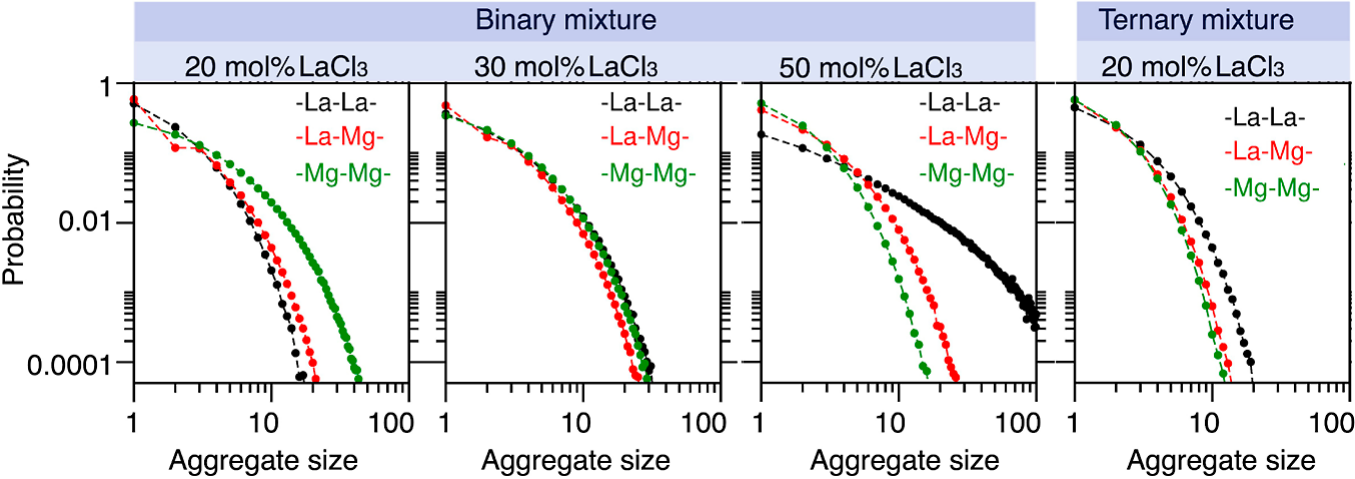
La–La, La–Mg, and Mg–Mg aggregate size distributions in binary and ternary mixtures calculated from the PIM using the AGGREGATES^62^ software by Bernardes. Notice how at equal 50% concentration in La^3+^ and Mg^2+^, La–La networks are significantly more prominent than others. In binary melts, only at 30 mol % concentration of La^3+^ we find that networks or aggregates have similar size distributions. Even at 20% LaCl_3_ in the ternary mixture, La^3+^ has the most network forming ability.

Figure 6 quantifies the probability of three idealized types of networks in our PIM simulations, namely, pure −La–La–, pure −Mg–Mg–, and hybrid −La–Mg– defined in terms of the nearest-neighbor distance cutoff of 4.9, 3.85, and 4.3 Å, respectively; these are the most-probable close-contact distances obtained from the location of the first peak of the respective pair distribution functions. Obviously, there are many other statistically relevant aggregate types that include La^3+^ and Mg^2+^ in patterns that are not a simple −La–Mg–La alternation. Yet, these three simple types of aggregates help us glean some interesting patterns as a function of LaCl_3_ concentration. For example, notice how massively larger the Cl^–^-decorated La^3+^ networks at 50 mol % LaCl_3_ are when compared with Mg^2+^ networks at identical concentration in binary mixtures. Notice also how similar the probability distributions are at 30% concentration of LaCl_3_. For the mixture melts we studied, only at 80% concentration of MgCl_2_ we observe aggregates of Mg^2+^ becoming larger than those of La^3+^. In the case of the ternary mixture, whereas all three types of aggregates are small, it is again those of La^3+^ that are more dominant even though the LaCl_3_ concentration is only 20% by mol compared to 30% of MgCl_2_. This highlights the distinct network forming ability of different multivalent cations with La^3+^ being the strongest in this study.

### 2.3 Mechanisms of Counterion Exchange

**Figure 7:**
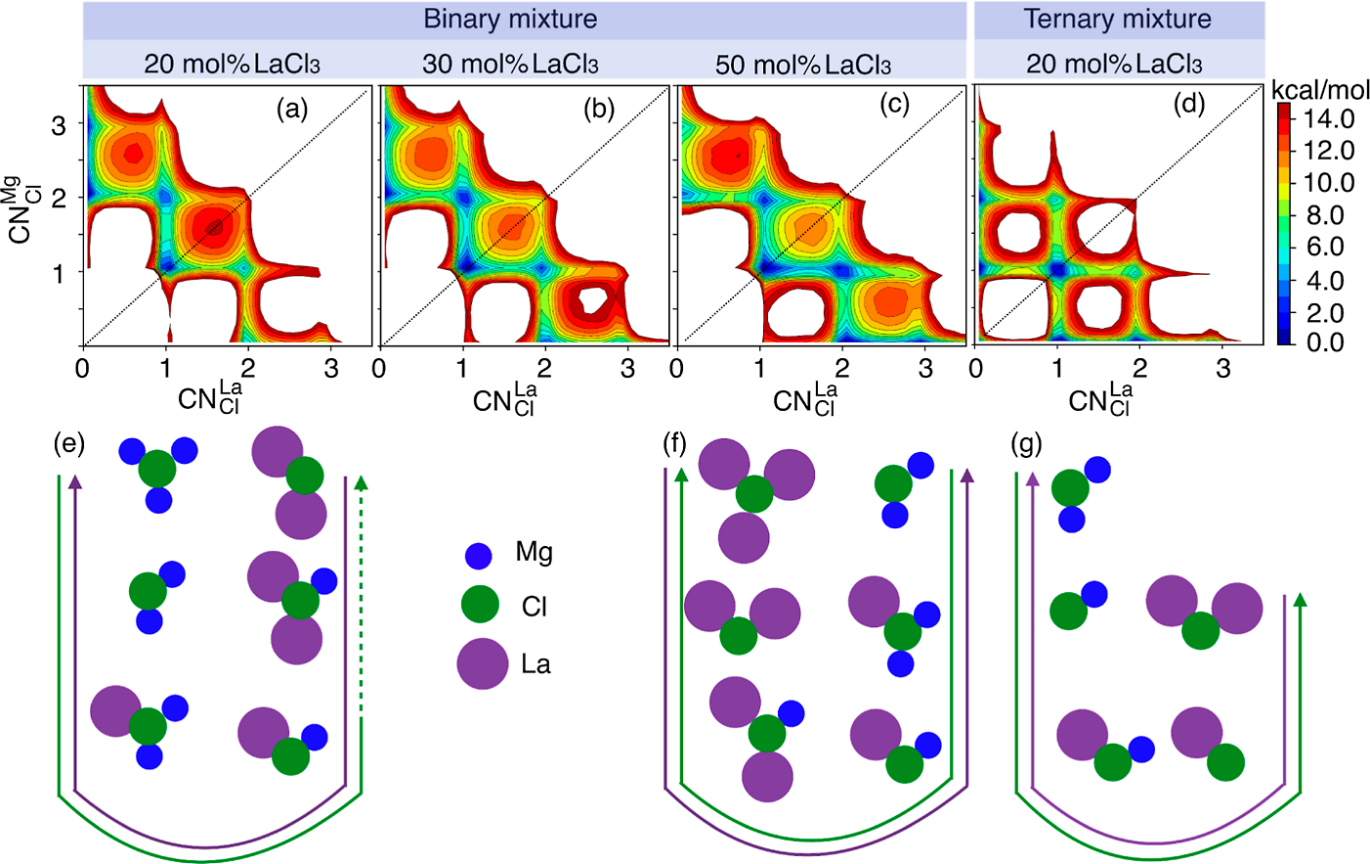
2D free energy surfaces as a function of the number of multivalent cations coordinating a single Cl^–^ ion (, ). Subfigures (a–c) show results for different concentrations in binary melts, and (d) shows results for the ternary melt. Mechanisms of cation exchange are defined by vertical and horizontal moves between minima in subfigures (a) through (d) which are pictorially depicted in subfigures (e–g). Likely pathways between fully Mg^2+^-coordinated and fully La^3+^-coordinated environments are depicted with green; opposite transitions are shown in purple. Dashed portions of the green mechanism in (e) indicate transitions of low probability for the 20 mol % LaCl_3_ in the binary mixture which become more probable for the 30 mol % melt. Diagonal lines in (a–d) are to guide the eye in recognizing symmetry in cationic coordination environments which are present in the ternary mixture and to some extent in the binary mixture at 30 mol % LaCl_3_. Results presented here are for the PIM; corresponding free energies from AIMD simulations are shown in Figure S5.

Through which mechanism does a Cl^–^ ion originally shared only by La^3+^ ions end up shared by Mg^2+^ ions or a combination of Mg^2+^ and La^3+^? It does so through a set of stepwise processes which can be derived from the joint probability distribution of  and  (the cationic coordination numbers of Cl^–^) and the concomitant free energy landscape associated with it. Consider the top panel in Figure 7 where such free energy lanscapes are depicted (very similar results from AIMD are presented in Figure S5). Notice how in each case “diagonal” displacements from the location of a minimum to that of a separate minimum are through high barriers whereas lateral or vertical moves are much more facile. These vertical and lateral moves will define the mechanistic steps for Cl^–^ exchange (*vide infra*).

To better understand Figure 7, we focus first on panel a corresponding to the 20 mol % LaCl_3_ in its binary mixture with MgCl_2_. In this figure, the purely Mg^2+^-bound Cl^–^ states are those with  = 0 and  = 2 or 3. States in which Cl^–^ is shared by La^3+^ and Mg^2+^ correspond to  = 1 and  = 1 or 2 as well as the low probability  = 2 and  = 1. The only La^3+^-bound state is the low probability  = 2 and  = 0.

It must be that the transition from the Mg^2+^-bound state with  = 3 to the only La^3+^-bound state in Figure 7a initiates through the reduction in the magnesium coordination number from 3 to 2, which creates a vacancy allowing the chloride ion to accept one La^3+^ ion, followed by the detachment of another Mg^2+^ ion creating the ( = 1,  = 1) state. The path to La^3+^-bound can then occur through the attachment of another La^3+^ ion and as a last step the detachment of the last remaining Mg^2+^ ion. Notice that each of these sequential steps are horizontal or vertical in the free energy profile; diagonal steps in which two exchanges occur simultaneously are free-energetically unfavorable. The reversible mechanism we just described is pictorially depicted in Figure 7e. The same mechanism persists at the 30 mol % LaCl_3_ concentration, but in this case La^3+^-bound states ( = 2 or 3,  = 0) are more probable as reflected by their lower free energies in Figure 7b.

The 50 mol % LaCl_3_ system with free energy depicted in Figure 7c is one with lanthanum-rich coordination environments around Cl^–^. This can be gleaned from the deep minima as a function of  with  = 0. The mechanism for going from the pure La^3+^ coordination of Cl^–^ to the one in which Mg^2+^ dominates is again stepwise and depicted in Figure 7f. In the case of the ternary mixture, the maximum number of Mg^2+^ or La^3+^ neighbors around a chloride ion reduces to 2 with the ion exchange mechanism depicted in Figure 7g. It is interesting that the top and bottom sides of the diagonal line in the 2D free energy surfaces are quite symmetrical in the ternary mixture and in the binary mixture with the 30 mol % LaCl_3_. This means that, around a chloride ion, the expected magnesium coordination environment for a given lanthanum coordination environment is the same as the expected lanthanum coordination environment for a given magnesium coordination environment.

## 3 Conclusions

For complex mixtures of molten salts of significant relevance to energy technologies, we found that different salt components play significantly different structural roles. The multivalent cations are network formers, and NaCl acts as a spacer salt between networks or aggregates. This has clear signatures in X-ray scattering where two different patterns of ion alternation can be detected; one of these is associated with prototypical charge alternation and the other with intermediate range order. In a way, this is not too different from the intermediate range order in ionic liquids where apolar domains separate charge networks (in our case NaCl separates multivalent cationic aggregates), except from the fact that the apolar domains in ILs are tethered to the network.

The coordination of multivalent cations should be thought of as an ensemble of concentration- and temperature-dependent states, not as a number. Furthermore, these states are coupled; more specifically, highly charged, highly polarizing cations such as La^3+^ affect the behavior of Cl^–^ anions in a way that changes the Cl^–^ coordination numbers of all cations. The higher the concentration of La^3+^, the higher the average Cl^–^ coordination numbers of all the cations; in addition, La^3+^ ions are also better network formers compared to Mg^2+^ ions.

This study was able to identify the mechanisms of ion exchange as a Cl^–^ goes from a fully La^3+^-coordinated environment to a fully Mg^2+^-coordinated one and *vice versa*. In each case, multiple barriers must be crossed step-by-step in the free energy landscape. These barriers are never higher than a few *k*_*B*_*T* units at the temperature at which these salts are being studied. What this means is that structures in these melts are labile, there are no long-lasting complexes or networks, and anions are constantly exchanging cationic partners on a microscopic time scale.

## Data Availability

Data sets for this article are made available within 30 days of the official acceptance date of this article by the journal in the Zenodo repository under the Digital Object Identifier (DOI): 10.5281/zenodo.10856841.

## Supporting Information Available

Experimental section describing salt samples, the generation of experimental and computational X-ray structure functions, PIM simulation methods and details, AIMD simulation methods and details, and free energy calculation methods and details; X-ray S(q) in its full range; from AIMD simulations, free energies as a function of multivalent cation distance and the number of shared Na^+^ ions, free energy as a function of multivalent cation distance and the number of shared Cl^–^ ions, free energy as a function of the Cl^–^ coordination number of the cations, and 2D free energy surface as a function of the Mg^2+^ and La^3+^ coordination numbers for a Cl^–^ anion (PDF)
