# Supplementary material for: Heterogeneous Structure, Mechanisms of Counterion Exchange, and the Spacer Salt Effect in Complex Molten Salt Mixtures Including LaCl3
Source: J Phys Chem B. 2024 Apr 16;128(16):3972–80. doi: 10.1021/acs.jpcb.4c01429 (PMC11056984; doi:10.1021/acs.jpcb.4c01429)
Supplement: Supplementary file 1 — jp4c01429_si_001.pdf [file jp4c01429_si_001.pdf]

Supporting Information: Heterogeneous Structure, Mechanisms of Counterion Exchange, and the Spacer Salt Effect in Complex Molten Salt Mixtures Including $LaCl_3$

Matthew S. Emerson,[†] Alexander S. Ivanov,[*,‡] Leighanne C. Gallington,[¶] Dmitry S. Maltsev,[‡] Phillip Halstenberg,[‡,§] Sheng Dai,[‡,§] Santanu Roy,[*,‡] Vyacheslav S. Bryantsev,[*,‡] and Claudio J. Margulis[*,†]

†*Department of Chemistry, The University of Iowa, Iowa City, IA 52242, United States*

‡*Chemical Sciences Division, Oak Ridge National Laboratory, Oak Ridge, TN 37831, United States*

¶*X-ray Science Division, Argonne National Laboratory, Argonne, IL 60439, United States*

§*Department of Chemistry, University of Tennessee, Knoxville, TN 37996, United States*

E-mail: ivanova@ornl.gov; roys@ornl.gov; bryantsevv@ornl.gov; claudio-margulis@uiowa.edu

Contents

S.1 Experimental Section **S-3**

S.1.1 Salt Samples S-3

S.1.2 X-ray Structure Functions S-3

S.1.3 PIM Simulation S-4

S.1.4 AIMD Simulations S-6

S.1.5 Free Energy Calculations S-7

S.2 Supplemental Figures and Tables **S-9**

S.2.1 X-Ray S(q) (Full Range) S-9

S.2.2 AIMD 2D Free Energy Diagrams S-10

References **S-12**

S.1 Experimental Section

The following subsections describe experimental, computational, and theory methods used in our study.

S.1.1 Salt Samples

Sealed quartz ampules (99.99% purity) of anhydrous $LaCl_3$, NaCl, and $MgCl_2$ were purchased from Aldrich-APL and combined in appropriate amounts in a glovebox to make binary and ternary salt mixtures. Using well-established procedures described in previous studies,[S1–S3] we proceeded to fuse the salt mixtures together at approximately 800 °C under dynamic vacuum. Afterward, the resulting salt compositions were finely crushed and then introduced into separate thin-walled quartz capillaries (1.5 mm O.D., 0.010 mm wall thickness, Charles Supper). The capillaries were subsequently sealed near the top under vacuum conditions.

S.1.2 X-ray Structure Functions

High-energy X-ray total scattering measurements at 900 °C were performed at beamline 11-ID-B of the Advanced Photon Source (APS) with an incident X-ray wavelength of 0.2115 Å (58.6 keV). The diffracted X-rays were collected using a silicon-based area detector (Perkin Elmer XRD1621) with 200×200 micron pixels positioned 160 mm behind the sample. We used the GSAS II program[S4] for the calibration of the precise sample to detector distance, beam center, detector tilt and rotation; crystalline CeO_2 powder (NIST 674b) was used as the calibrant.

We collected X-ray scattering data for the molten samples in the furnace originally designed by Chupas et al.[S5] Additionally, we collected data for an empty quartz capillary at the same temperature to serve as a reference background signal subtraction. Integration of the 2D detector images to 1D diffraction patterns (q = 0.7-18 $Å^{-1}$) was done with the GSAS II software, using masks to remove the beamstop and dead pixels. Background signal subtraction (empty quartz capillary) and additional corrections (sample self-absorption, multiple scattering, and inelastic Compton scatter-

ing) were performed using the PDFgetX2[S6] software according to standard procedures.[S7,S8] Following these corrections, the resulting data were normalized with respect to the average electron density determined by the weighted sum of the corresponding ionic X-ray form factors, thereby yielding the total structure function, S(q);

$$\mathrm{S(q)} = \frac{I_{coh}(\mathrm{q}) - \sum_i \chi_i f_i^2(\mathrm{q})}{\left[\sum_i \chi_i f_i(\mathrm{q})\right]^2}. \tag{S.1}$$

In Equation S.1, q denotes the magnitude of the scattering vector ($\mathrm{q} = 4\pi\sin(\theta)/\lambda$), where 2θ is the scattering angle, and λ is the incident X-ray wavelength. χ_i and $f_i(\mathrm{q})$ are the molar fraction and q-dependent X-ray ionic form factor[S9] of species i; I_{coh} is the coherent scattering intensity. The total X-ray structure function can also be computed from molecular dynamics simulations using Equation S.2;

$$\mathrm{S(q)} = \frac{\rho_0 \sum_i \sum_j \chi_i \chi_j f_i(\mathrm{q}) f_j(\mathrm{q}) \int_0^\infty 4\pi r^2 \left(g_{ij}(r) - 1\right) \frac{\sin(\mathrm{q}r)}{\mathrm{q}r} dr}{\left[\sum_v \chi_v f_v(\mathrm{q})\right]^2} \tag{S.2}$$

where ρ_0 is the average ionic number density of the system and $g_{ij}(r)$ is the pair radial distribution function between species i and j. S(q) can be decomposed into subcomponents such that

$$\mathrm{S(q)} = \sum_i \sum_{j \geq i} S_{ij}(\mathrm{q}) \tag{S.3}$$

where

$$\mathrm{S_{ii}(q)} = \frac{\rho_0 \chi_i \chi_i f_i(\mathrm{q}) f_i(\mathrm{q}) \int_0^\infty 4\pi r^2 \left(g_{ii}(r) - 1\right) \frac{\sin(\mathrm{q}r)}{\mathrm{q}r} dr}{\left[\sum_v \chi_v f_v(\mathrm{q})\right]^2} \tag{S.4}$$

and

$$\mathrm{S_{ij}(q)} = 2 * \frac{\rho_0 \chi_i \chi_j f_i(\mathrm{q}) f_j(\mathrm{q}) \int_0^\infty 4\pi r^2 \left(g_{ij}(r) - 1\right) \frac{\sin(\mathrm{q}r)}{\mathrm{q}r} dr}{\left[\sum_v \chi_v f_v(\mathrm{q})\right]^2} \tag{S.5}$$

S.1.3 PIM Simulation

For all PIM simulations, thorough temperature equilibration protocols at 1 bar were applied and these have already been described in reference S10. The functional form of the PIM force field can also be found in Equations S3-S5 of the Supporting Information in reference S10. All PIM

simulations were carried out using the Metalwalls software[S11] with number of ions for each melt mixture provided in the column labeled PIM in Table S1.

Table S1: Number of Ions in PIM and AIMD Simulations

Salt	Simulation Type							
	PIM				AIMD			
	# La	#Na	#Mg	#Cl	# La	#Na	#Mg	#Cl
$LaCl_3$-NaCl (20-80)					20	80	0	140
$LaCl_3$-$MgCl_2$ (20-80)	300	0	1200	3300	20	0	80	220
$LaCl_3$-$MgCl_2$ (30-70)	300	0	700	2300				
$LaCl_3$-$MgCl_2$ (50-50)	500	0	500	2500	50	0	50	250
$LaCl_3$-NaCl-$MgCl_2$ (20-50-30)	200	500	300	1700	20	50	30	170

Production runs were 2 ns in duration at 1 bar and 900 °C using the Nosé-Hoover[S12,S13] thermostat and barostat with chain lengths of 5, and relaxation times of 500 fs and 2500 fs respectively as coded in Metalwalls release 20.05.[S11] Simulations used the Ewald summation[S11,S14,S15] with tolerances, dipole convergence threshold, and real space cutoffs as described in reference S10; notice that there are two cutoffs. The smaller one is for PIM trajectories used to pre-equilibrate snapshots to be later used in AIMD simulations (column AIMD in Table S1; *vide infra*).

Just as in prior studies using the PIM,[S1,S10,S16–S19] we treat multivalent cations as non-polarizable. Table S2 lists charges and ionic polarizabilities for each ion in our simulations, charge-dipole damping parameters are listed in Table S3, and BMH parameters are listed in Table S4. As discussed by Hutchinson and Ishii,[S16,S20] as well as in our prior work,[S10] repulsion parameters between cations have negligible contributions when compared with their Coulomb repulsion and it is common for cross dispersion parameters for cations to be approximated as the same (as examples see references S17 and S18). These considerations result in rows for Mg^{2+}-Na^{+} and Mg^{2+}-Mg^{2+} as well as for La^{3+}-Mg^{2+} and La^{3+}-$Na^{+}$to be the same in Table S4.

Table S2: Ionic polarizabilies and charges

Ion	q(e)	α (Bohr^3))
Na^+	+1.000	0.9[S20]
Mg^{2+}	+2.000	0[S1]
La^{3+}	+3.000	0[S17]
Cl^-	-1.000	20.0[S20]

Table S3: Charge-Dipole Damping Parameters

Ion-Pair	b_4^{ij}=b_4^{ji}	c_4^{ij}	c_4^{ji}	Ref.
Na^+-Cl^-	1.760	3.000	0.697	S20
Mg^{2+}-Cl^-	1.873	2.875	N/A	S1,S19
La^{3+}-Cl^-	1.258	1.000	N/A	S18

Table S4: BMH Parameters

Ion-Pair	α_{ij}	B_{ij}	C_{ij}^6	C_{ij}^8	b_{ij}^6	b_{ij}^8	Ref.
La^{3+}-La^{3+}	3.000	15.0	47.70	100.0	1.50	1.00	S18
La^{3+}-Na^+	3.000	10.0	34.8	94.6	1.50	1.00	S10
La^{3+}-Mg^{2+}	3.000	10.0	34.8	94.6	1.50	1.00	*
Na^+-Na^+	5.000	1.0	11.7	51.8	1.70	1.70	S20
Mg^{2+}-Na^+	5.000	1.0	0.0	0.0	1.70	1.70	*
Mg^{2+}-Mg^{2+}	5.000	1.0	0.0	0.0	1.70	1.70	S1,S19
La^{3+}-Cl^-	1.800	450.0	97.22	600.0	1.50	1.00	S18
Mg^{2+}-Cl^-	1.732	68.6	10.0	20.0	1.70	1.70	S1,S19
Na^+-Cl^-	1.726	67.5	47.4	187.3	1.70	1.70	S20
Cl^--Cl^-	1.797	275.1	140.0	280.0	1.70	1.70	S20

S.1.4 AIMD Simulations

Starting points for all AIMD simulations were frames selected from equilibrated PIM simulations with number of ions provided in Table S1 and volume equal to the average value from the corresponding PIM run. AIMD simulations were carried out in the constant volume and temperature ensemble (NVT) using a time step of 1 fs. The temperature was kept at 900 °C using the Nosé-Hoover chain thermostat[S21] with time constant for velocity rescaling set to 1.0 ps. The last 60 ps of trajectories that were at least 100 ps in duration were used for computing S(q) and free energies.

Simulations were carried out using the Quickstep module of the CP2K 6.1 package[S22,S23] using the PBE exchange-correlation functional[S24–S27] and D3 dispersion correction by Grimme.[S28] All ions used the MOLOPT basis set[S29] of triple zeta valence plus polarization (TZVP-MOLOPT) together with Goedecker-Teter-Hutter (GTH) pseudopotentials.[S30] La was treated using a standard GTH-PBE pseudopotential involving 11 valence electrons (5s25p65d16s2) along with the corresponding TZVP-MOLOPT-SR-GT basis set. Cutoffs were set to 800 Ry for the plane wave basis (CUTOFF) and 80 Ry for a reference grid (REL_CUTOFF). For an optimized convergence of SCF cycles we used the orbital transformation method with the FULL_ALL preconditioner and a conjugate gradient minimizer.

S.1.5 Free Energy Calculations

A significant portion of the structural analysis in real space and our prediction of mechanisms of ion exchange is done using the free energy formalisms we describe in this section. If we define r_i as the distance between the ith Cl^- and a cation, and $r^\dagger$ as the distance from the cation to the boundary of its first Cl^- coordination shell, then the chloride coordination number of that cation ($CN_{Cat}^{\mathrm{Cl}^-}$) is defined via smooth functions $f_i^{\mathrm{Cl}^-}$ as

$$CN_{Cat}^{\mathrm{Cl}^-} = \sum_{i=1}^{N_{\mathrm{Cl}^-}} f_i^{\mathrm{Cl}^-} = \sum_{i=1}^{N_{\mathrm{Cl}^-}} \frac{1-\left(\frac{r_i}{r^\dagger}\right)^{12}}{1-\left(\frac{r_i}{r^\dagger}\right)^{24}}. \tag{S.6}$$

In Equation S.6, *Cat* stands for a given La^{3+}, Mg^{2+}, or Na^+ ion; $r^\dagger$ is obtained from the first minimum after the first peak in the *Cat*-Cl^- $g(r)$, and N_{Cl^-} is the total number of chloride ions. We have shown in prior publications that the powers (12,24) in the equation for f_i result in smooth and correct values of CN. Analogously, the cationic coordination number of a Cl^- ion ($CN_{\mathrm{Cl}^-}^{Cat}$, i.e.,

the number of La^{3+} or Mg^{2+} ions around a Cl^- ion) is expressed as

$$CN^{Cat}_{Cl^-} = \sum_{i=1}^{N_{Cat}} f_i^{Cat} = \sum_{i=1}^{N_{Cat}} \frac{1-\left(\frac{r_i}{r^\dagger}\right)^{12}}{1-\left(\frac{r_i}{r^\dagger}\right)^{24}} \tag{S.7}$$

where N_{Cat} is the total number of La^{3+} or Mg^{2+} ions. From these coordination numbers one can establish probability distributions, $P(CN)$, and from the probability distributions, free energies $W(CN) = -k_B\ T\ ln[P(CN)]$, where T is the system temperature and k_B is the Boltzmann constant. We can also define joint probability distributions $P(CN^{La^{3+}}_{Cl^-}, CN^{Mg^{2+}}_{Cl^-})$ and corresponding free energies $W(CN^{La^{3+}}_{Cl^-}, CN^{Mg^{2+}}_{Cl^-}) = -k_B\ T\ ln[P(CN^{La^{3+}}_{Cl^-}, CN^{Mg^{2+}}_{Cl^-})]$. These allow us to study mechanisms of anion migration between lanthanum-rich and magnesium-rich environments.

In equation S.6, $f_i^{Cl^-}$ is the contribution of the ith Cl^- ion to the coordination structure of a cation. If we add an extra label to $f_i^{Cl^-}$ denoting the index of said cation, we have that $f_{ij}^{Cl^-}$ and $f_{ik}^{Cl^-}$ are the contributions of the ith Cl^- to the coordination environments of the jth and k^{th} cations, respectively. We can compute the number of Cl^- ions shared by the two cations as

$$n^{Cl^-}_{Cat} = \sum_{i=1}^{N_{Cl^-}} f_{ij} f_{ik}. \tag{S.8}$$

$n^{Cl^-}_{Cat}$ can now be used as a variable with a given probability distribution from which a free energy can be computed. For example, Figure 4 shows free energies as a function of the distance between cations and $n^{Cl^-}_{Cat}$. An analogous variable, $n^{Na^+}_{Cat}$, can be used to describe the number of Na^+ ions shared by cations. Such variable is used in Figure 2.

S.2 Supplemental Figures and Tables

S.2.1 X-Ray S(q) (Full Range)

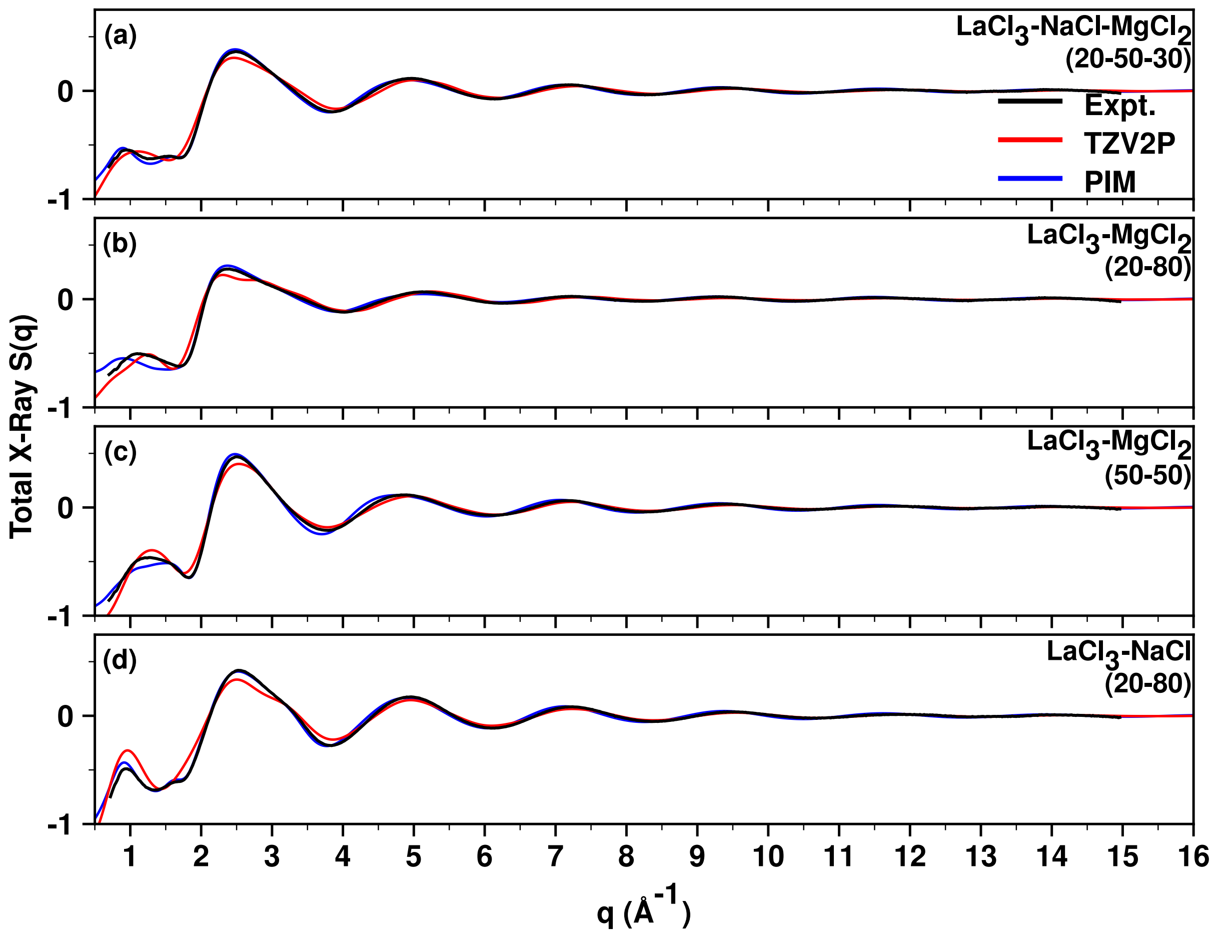

Figure S1: Comparison of structure functions S(q) at 900 °C for $LaCl_3$-$MgCl_2$, and $LaCl_3$-NaCl-$MgCl_2$ mixtures with mole% denoted in figure titles. Black, red, and blue lines correspond to experimental, AIMD, and PIM results respectively. PIM and experimental S(q) for subfigure (d) use the same data as in reference S10.

S.2.2 AIMD 2D Free Energy Diagrams

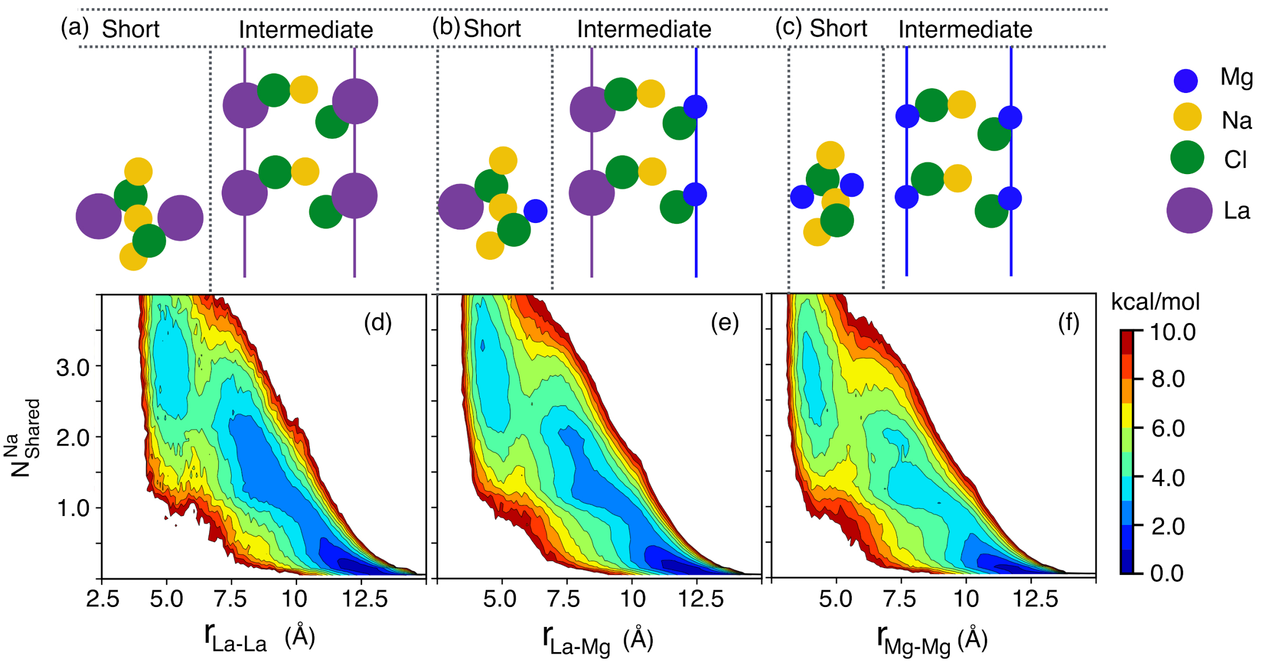

Figure S2: For the $LaCl_3$-NaCl-$MgCl_2$ (20%-50%-30%) ternary mixture, (a) through (c), schemes depicting short and intermediate-range structural motifs associated with the free energies as a function of La-La, La-Mg, and Mg-Mg separation distances and the number of shared Na^+ ions between them plot in subfigures (d) through (f). Results presented here are for AIMD simulations.

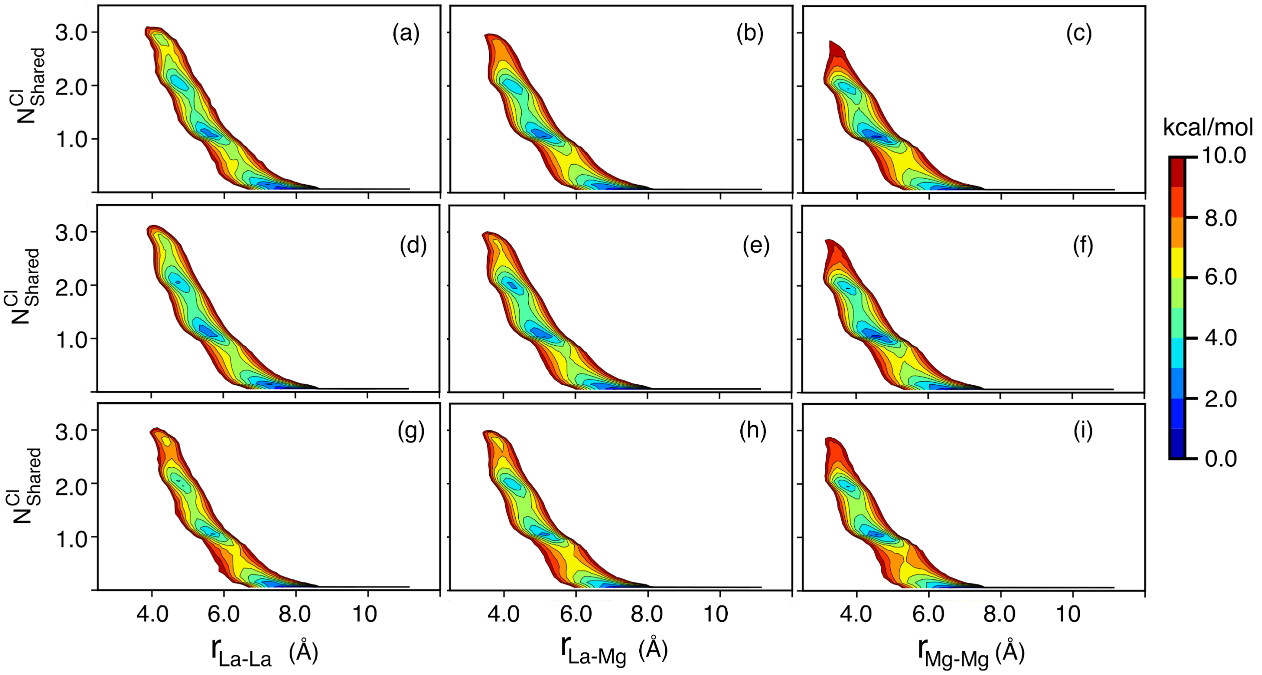

Figure S3: For 20 mole% (a-c), 50 mole% (d-f) $LaCl_3$ in binary $LaCl_3$-$MgCl_2$ mixture melts and for the 20 mole% (g-i) $LaCl_3$ in the ternary $LaCl_3$-NaCl-$MgCl_2$ melt; 2D-free energy surfaces as a function of the number of shared chlorides between multivalent cations and their corresponding distance. Results presented here are for AIMD simulations.

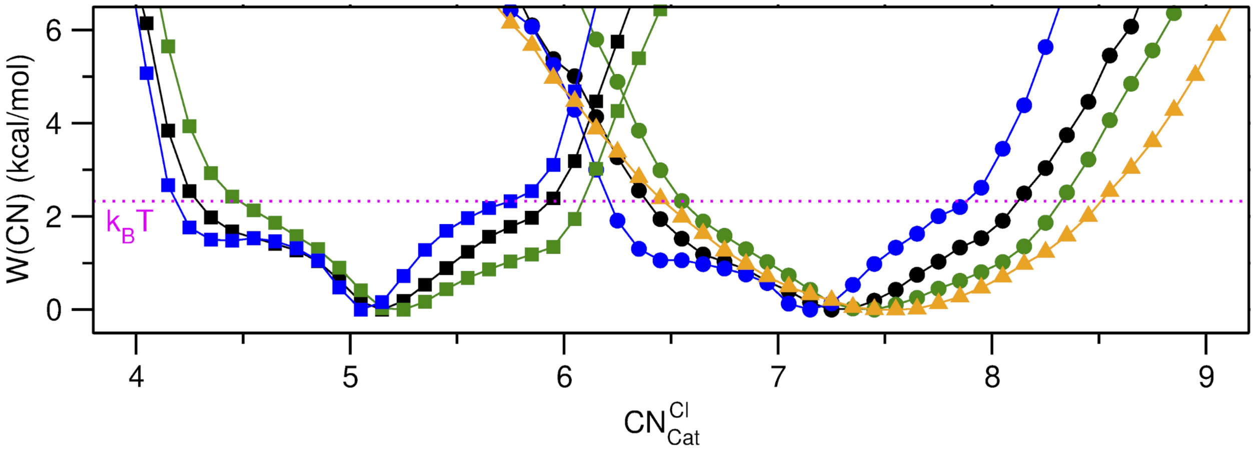

Figure S4: For all cations, free energy as a function of their Cl^- coordination number. These are depicted with circles for La^{3+} squares for Mg^{2+}, and triangles for Na^+. Different colors indicate different concentrations; specifically, in binary $LaCl_3$-$MgCl_2$ mixtures black is for 20 mole% $LaCl_3$, and green is for 50 mole% $LaCl_3$. In the ternary mixture, which is 20 mole% in $LaCl_3$, free energies for La^{3+} and Mg^{2+} are shown in blue and that for Na^+ in yellow. The thermal energy, which is high compared to several of the free energy minima ($k_B T$ = 2.33 kcal/mol), is indicated with a horizontal magenta line. Results presented here are for AIMD.

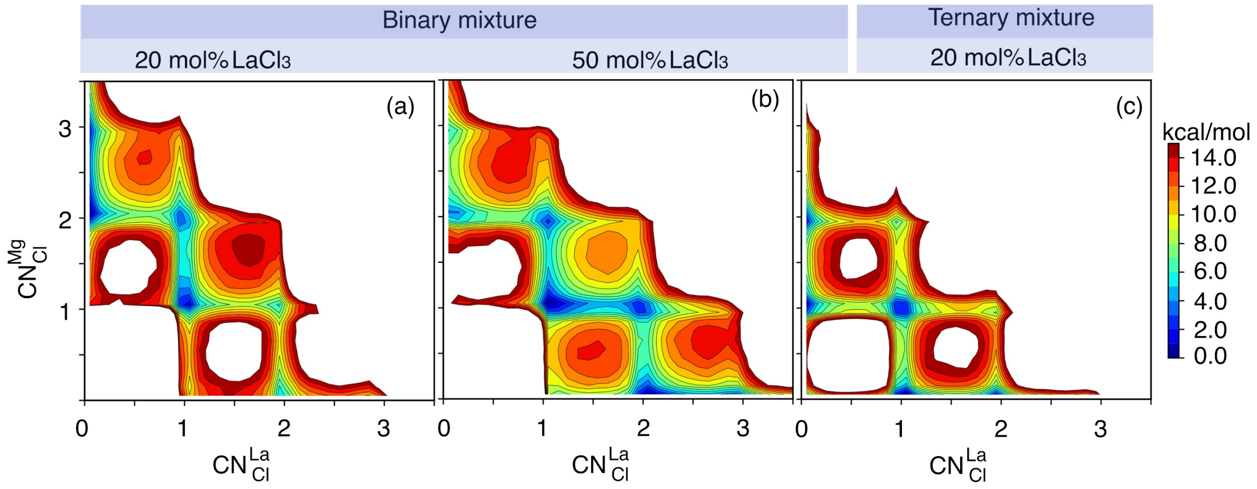

Figure S5: 2D-free energy surfaces as a function of the number of multivalent cations coordinating a single Cl^- ion ($CN_{Cl^-}^{La^{3+}}$, $CN_{Cl^-}^{Mg^{2+}}$). Subfigures a,b show results for different concentrations in binary melts and subfigure c shows results for the ternary melt. Results presented here are from AIMD simulations.

References

(S1) Wu, F.; Roy, S.; Ivanov, A. S.; Gill, S. K.; Topsakal, M.; Dooryhee, E.; Abeykoon, M.; Kwon, G.; Gallington, L. C.; Halstenberg, P.; Layne, B.; Ishii, Y.; Mahurin, S. M.; Dai, S.; Bryantsev, V. S.; Margulis, C. J. Elucidating ionic correlations beyond simple charge alternation in molten $MgCl_2$–KCl mixtures. *J. Phys. Chem. Lett.* **2019**, *10*, 7603–7610.

(S2) Roy, S.; Brehm, M.; Sharma, S.; Wu, F.; Maltsev, D. S.; Halstenberg, P.; Gallington, L. C.; Mahurin, S. M.; Dai, S.; Ivanov, A. S.; Margulis, C. J.; Bryantsev, V. S. Unraveling Local Structure of Molten Salts via X-ray Scattering, Raman Spectroscopy, and Ab Initio Molecular Dynamics. *J. Phys. Chem. B* **2021**, *125*, 5971–5982.

(S3) Roy, S.; Sharma, S.; Karunaratne, W. V.; Wu, F.; Gakhar, R.; Maltsev, D. S.; Halstenberg, P.; Abeykoon, M.; Gill, S. K.; Zhang, Y.; Mahurin, S. M.; Dai, S.; Bryantsev, V. S.; Margulis, C. J.; Ivanov, A. S. X-ray scattering reveals ion clustering of dilute chromium species in molten chloride medium. *Chem. Sci.* **2021**, *12*, 8026–8035.

(S4) Toby, B. H.; Von Dreele, R. B. GSAS-II: the genesis of a modern open-source all purpose crystallography software package. *J. Appl. Crystallogr.* **2013**, *46*, 544–549.

(S5) Chupas, P. J.; Chapman, K. W.; Kurtz, C.; Hanson, J. C.; Lee, P. L.; Grey, C. P. A versatile sample-environment cell for non-ambient X-ray scattering experiments. *J. Appl. Crystallogr.* **2008**, *41*, 822–824.

(S6) Qiu, X.; Thompson, J. W.; Billinge, S. J. L. PDFgetX2: a GUI-driven program to obtain the pair distribution function from X-ray powder diffraction data. *J. Appl. Crystallogr.* **2004**, *37*, 678–678.

(S7) Fischer, H. E.; Barnes, A. C.; Salmon, P. S. Neutron and X-ray Diffraction Studies of Liquids and Glasses. *Rep. Prog. Phys.* **2006**, *69*, 233–299.

(S8) Billinge, S. J.; Egami, T. *Underneath the Bragg Peaks*; Elsevier, 2012; Vol. 16.

(S9) Brown, P. J.; Fox, A. G.; Maslen, E. N.; O'Keefe, M. A.; Willis, B. T. M. *International Tables for Crystallography*; International Tables for Crystallography; 2006; Chapter Chapter 6.1, pp 554–595.

(S10) Emerson, M. S.; Sharma, S.; Roy, S.; Bryantsev, V. S.; Ivanov, A. S.; Gakhar, R.; Woods, M. E.; Gallington, L. C.; Dai, S.; Maltsev, D. S.; Margulis, C. J. Complete Description of the $LaCl_3$–NaCl Melt Structure and the Concept of a Spacer Salt That Causes Structural Heterogeneity. *J. Am. Chem. Soc.* **2022**, *144*, 21751–21762.

(S11) Marin-Laflèche, A.; Haefele, M.; Scalfi, L.; Coretti, A.; Dufils, T.; Jeanmairet, G.; Reed, S.; Serva, A.; Berthin, R.; Bacon, C.; Bonella, S.; Rotenberg, B.; Madden, P.; Salanne, M. MetalWalls: A classical molecular dynamics software dedicated to the simulation of electrochemical systems. *J. Open Source Softw.* **2020**, *5*.

(S12) Nosé, S. A unified formulation of the constant temperature molecular dynamics methods. *J. Chem. Phys.* **1984**, *81*, 511–519.

(S13) Hoover, W. G. Canonical dynamics: Equilibrium phase-space distributions. *Phys. Rev. A* **1985**, *31*, 1695–1697.

(S14) Ewald, P. P. Die Berechnung optischer und elektrostatischer Gitterpotentiale. *Ann. Phys.* **1921**, *369*, 253–287.

(S15) Aguado, A.; Madden, P. A. Ewald summation of electrostatic multipole interactions up to the quadrupolar level. *J. Chem. Phys.* **2003**, *119*, 7471–7483.

(S16) Hutchinson, F.; Wilson, M.; Madden, P. A. A unified description of MCl_3 systems with a polarizable ion simulation model. *Mol. Phys.* **2001**, *99*, 811–824.

(S17) Glover, W. J.; Madden, P. A. Raman spectra of ionic liquids: A simulation study of $LaCl_3$ and its mixtures with alkali chlorides. *J. Chem. Phys.* **2004**, *121*, 7293–7303.

(S18) Salanne, M.; Simon, C.; Turq, P.; Madden, P. A. Calculation of Activities of Ions in Molten Salts with Potential Application to the Pyroprocessing of Nuclear Waste. *J. Phys. Chem. B* **2008**, *112*, 1177–1183.

(S19) Wu, F.; Sharma, S.; Roy, S.; Halstenberg, P.; Gallington, L. C.; Mahurin, S. M.; Dai, S.; Bryantsev, V. S.; Ivanov, A. S.; Margulis, C. J. Temperature dependence of short and intermediate range order in molten $MgCl_2$ and its mixture with KCl. *J. Phys. Chem. B* **2020**, *124*, 2892–2899.

(S20) Ishii, Y.; Kasai, S.; Salanne, M.; Ohtori, N. Transport coefficients and the Stokes–Einstein relation in molten alkali halides with polarisable ion model. *Mol. Phys.* **2015**, *113*, 2442–2450.

(S21) Nosé, S. A unified formulation of the constant temperature molecular dynamics methods. *J. Chem. Phys.* **1984**, *81*, 511–519.

(S22) Vandevondele, J.; Krack, M.; Mohamed, F.; Parrinello, M.; Chassaing, T.; Hutter, J. Quickstep: Fast and accurate density functional calculations using a mixed Gaussian and plane waves approach. *Comput. Phys. Commun.* **2005**, *167*, 103–128.

(S23) Hutter, J.; Iannuzzi, M.; Schiffmann, F.; VandeVondele, J. CP2K: atomistic simulations of condensed matter systems. *WIREs Comput. Mol. Sci.* **2014**, *4*, 15–25.

(S24) Perdew, J. P.; Burke, K.; Ernzerhof, M. Generalized Gradient Approximation Made Simple. *Phys. Rev. Lett.* **1996**, *77*, 3865–3868.

(S25) Zhang, Y.; Yang, W. Comment on “Generalized Gradient Approximation Made Simple”. *Phys. Rev. Lett.* **1998**, *80*, 890–890.

(S26) Perdew, J. P.; Burke, K.; Ernzerhof, M. Comment on "Generalized Gradient Approximation Made Simple" - Reply. *Phys. Rev. Lett.* **1998**, *80*, 891–891.

(S27) Perdew, J. P.; Ruzsinszky, A.; Csonka, G. I.; Vydrov, O. A.; Scuseria, G. E.; Constantin, L. A.; Zhou, X.; Burke, K. Restoring the Density-Gradient Expansion for Exchange in Solids and Surfaces. *Phys. Rev. Lett.* **2008**, *100*.

(S28) Grimme, S.; Antony, J.; Ehrlich, S.; Krieg, H. A consistent and accurate ab initio parametrization of density functional dispersion correction (DFT-D) for the 94 elements H-Pu. *J. Chem. Phys.* **2010**, *132*, 154104.

(S29) Vandevondele, J.; Hutter, J. Gaussian basis sets for accurate calculations on molecular systems in gas and condensed phases. *J. Chem. Phys.* **2007**, *127*, 114105.

(S30) Goedecker, S.; Teter, M.; Hutter, J. Separable dual-space Gaussian pseudopotentials. *Phys. Rev. B* **1996**, *54*, 1703–1710.
